# Regulatory effects of hawthorn leaf flavonoids and stevioside on the uterine function and eggshell quality in laying hens

**DOI:** 10.1016/j.aninu.2025.06.007

**Published:** 2025-08-26

**Authors:** Jiao Wang, Qiang Jiang, Xianlei Li, Xianghong Zhao, Yuming Guo, Bingkun Zhang, Zhonghua Ning, Zengpeng Lv

**Affiliations:** a State Key Laboratory of Animal Nutrition and Feeding, College of Animal Science and Technology, China Agricultural University, Beijing 100193, China; b Beijing SinoAgri Uplus Biotech Co., Ltd., Beijing 100194, China; c China Agricultural University-Sichuan Advanced Agricultural & Industrial Institute, Chengdu 611430, Sichuan, China

**Keywords:** Plant extract, Uterine function, Eggshell quality, Transcriptome, Microbiota

## Abstract

This study aimed to investigate the effects of hawthorn leaf flavonoids (HF) and stevioside (ST) on eggshell quality, uterine function of the oviduct, and microbial composition in laying hens. A total of 630 Jing Tint 6 laying hens aged 66 weeks with similar body weights (1.8 ± 0.2 kg) were randomly divided into 7 groups with 6 replicates of 15 hens each. The control group (CON) was fed a basal diet, while the treatment groups were supplemented with 120 mg/kg HF (HF120), 120 mg/kg ST (ST120), 120 mg/kg HF + 120 mg/kg ST (HF120+ST120), 120 mg/kg HF + 60 mg/kg ST (HF120+ST60), 60 mg/kg HF + 120 mg/kg ST (HF60+ST120), and 60 mg/kg HF + 60 mg/kg ST (HF60+ST60) for 8 weeks. The results indicated that the combined dietary supplementation of HF and ST significantly improved eggshell strength, thickness, and fracture toughness (*P* < 0.05), with the greatest enhancement observed in the HF120+ST120 group. The CON, HF120, ST120, and HF120+ST120 groups were selected for further analyses. All treatments significantly increased the total thickness of the eggshell ultrastructure (*P* = 0.003). Particularly, the HF120+ST120 group significantly increased the frequency of in mammilla early fusion (*P* = 0.001), and reduced both the frequency of B-type mammillae (*P* = 0.034) and late fusion (*P* = 0.008). Serum calcium levels were significantly higher in the HF120+ST120 group compared to the CON group (*P* = 0.020). Histological analysis revealed that dietary supplementation with HF and ST enhanced the duodenal structure (increased villus height and villus-to-crypt ratio) and uterine structure (increased villus fold height), thereby promoting calcium absorption (*P* < 0.05). Furthermore, the HF120+ST120 treatment significantly increased antioxidant capacity and levels of anti-inflammatory cytokines (interleukin-4 and interleukin-10) in the uterus compared to the CON group (*P* < 0.05). Transcriptomic analysis of the uterus showed that the HF120+ST120 treatment influenced the expression of genes involved in calcium transport and matrix protein synthesis, including 13 transporter genes related to eggshell mineralization (*CALB1*, *ATP2B1*, *CA4*, etc.) and 5 genes encoding matrix proteins (*MEPE*, *BPIFB3*, *SPP1*, *SPP1*, and *CLU*; *P* < 0.05). Additionally, the abundance of Proteobacteria and *Mycoplasma* was significantly reduced in the HF120+ST120 treatment group (*P* < 0.05), with *Rikenellaceae_RC9_gut_group* and *Ruminococcus* dominating in this group. In conclusion, dietary supplementation with HF and ST could improve the intestinal and uterine villus morphology, enhance uterine antioxidant and anti-inflammatory capability, balance microecology, and improve calcium transport and matrix protein synthesis in the uterus, ultimately improving the eggshell quality of laying hens. This study provides a novel strategy for improving eggshell quality using plant extract combination, which may help to reduce economic losses in laying hen farming.

## 1 Introduction

Egg is a rich source of fatty acids, vitamins, minerals, and high-quality protein, all essential for human health. However, eggshell cracking significantly reduces both the nutritional value and economic efficiency of eggs, particularly during the late laying period ([Bibr bib45][Bibr bib48]). This decline is likely due to prolonged laying, which leads to oviduct aging, metabolic disorders, and shell gland hypoplasia, resulting in a substantial decrease in eggshell quality (Bain et al., 2013). Furthermore, oxidative stress and inflammatory responses are critical factors that affect oviduct health and eggshell quality (Liu et al., 2023a). Therefore, developing effective nutritional strategies to regulate oviduct function and enhance eggshell quality is crucial for improvingfeeding efficiency and extending the shelf life of egg products.

Eggshell quality is influenced by complex factors, and its formation is precisely regulated by genetic and biological pathways, reflecting the interaction between organic and inorganic components (Qiu et al., 2020). Mineralization involves the transport, regulation, and collaboration of multiple ion carriers to form a multilevel ultrastructure under the regulatory influence of calcium carbonate matrix proteins in uterine fluid (Wang et al., 2020). Transcriptomic analysis of the uterus revealed that aging alters the expression of calcium transporter genes, affecting calcium uptake and transport processes (Feng et al., 2020). Moreover, the ability to metabolize calcium was found to be positively correlated with eggshell strength and thickness (Jin et al., 2024). Calcium ions (Ca^2+^) required for eggshell formation are primarily absorbed from the diet via the duodenum, and changes in intestinal permeability, morphology, and structure can affect nutrient absorption. This is often reflected in a significant decrease in calcium ion absorption and transport efficiency in older laying hens (Jin et al., 2024). The structure and composition of the intestinal microbiota play a crucial role in immune system development, and its dysbiosis can negatively impact hen health and performance (Ruesga-Gutiérrez et al., 2022). Furthermore, poultry gut microbiota can be transmitted to the reproductive tract, playing a beneficial role in egg production, egg quality, and reproductive tract health in laying hens (Cheng et al., 2024; Gao et al., 2025).

Hawthorn leaf flavonoids (HF) are bioactive compounds extracted from hawthorn leaves, exhibiting a range of beneficial properties, including anti-inflammatory, antioxidant, and antibacterial effects (Dai et al., 2024). It is demonstrated that supplementing the aged breeder hens with dietary HF improved the laying performance and alleviated oxidative stress in the reproductive organs (Dai et al., 2021). Similarly, supplementing genistein in the diets of laying hens can significantly improve the eggshell strength and thickness (Lv et al., 2018, 2019). Stevioside (ST), a diterpene glycoside derived from stevia, is metabolized by intestinal microorganisms into steviol, which is then absorbed by the body (Jiang et al., 2022). Stevioside has been shown to regulate various biological functions, including anti-inflammatory activities, oxidative stress alleviation, and calcium metabolism regulation (Jiang et al., 2021). Dietary ST supplementation for aged breeder hens could improve egg production and eggshell quality by enhancing calcium content and antioxidant capacity in the uterus (Jiang et al., 2020). Furthermore, stevia extract has been found to alter the gut microbiota composition in laying hens, increasing microbial diversity, which in turn positively impacts overall health and production performance (Wen et al., 2024).

Recent studies have primarily focused on investigating the effects of individual plant extract additives on eggshell quality. Compared to other flavonoids, the cost of HF is significantly lower. Moreover, the combined effects of HF and ST on the health of laying hens and eggshell quality appear to be complementary and cost-effectiveness. Therefore, the present study utilized transcriptomic and microbiomics techniques to investigate the regulatory mechanisms of HF and ST supplementation on eggshell quality and uterine function in the oviduct of laying hens, providing a scientific basis for the practical application of HF and ST as feed additives.

## 2 Materials and methods

### 2.1 Animal ethics statement

The experimental animal procedures in this study were conducted at the Meat Chicken Science and Technology Backyard in Dongchengfang (Zhuozhou, Hebei, China) and were approved by the Animal Care and Use Committee of China Agricultural University (approval number: AW02105202-1-1).

### 2.2 Experiment materials

Hawthorn leaf flavonoids was obtained from Nantong Feiyu Biotechnology Co., Ltd. (Nantong, Jiangsu, China), with a purity of 80%. A Vanquish UHPLC system was coupled to a Q-Exactive HF-X hybrid quadrupole orbital rap mass spectrometer (Thermo Fisher Scientific Inc., Waltham, MA, USA) for the detection of small molecular compounds in HF. The seven major bioactive compounds and their respective proportions were vitexin-2-O-rhamnoside (14.27%), keracyanin rhamnoside (5.62%), epicatechin (2.75%), hyperoside (2.52%), chlorogenic acid (2.39%), vitexin (1.96%), and quercetin (0.96%). Stevioside, obtained from Shaanxi Yihaochuan Biotechnology Co., Ltd. (Xi'an, Shanxi, China), had a total glycoside content of 95%.

### 2.3 Experiment design

A total of 630 healthy laying hens (Jing Tint 6) aged 66 weeks were randomly divided into 7 groups with 6 replicates of 15 hens each. To ensure consistent egg production across all groups before the formal trial, a 2-week pre-feeding was conducted during which all hens were fed a standard corn-soybean meal basal diet. At the start of the formal 8-week experiment, the control group (CON) continued to receive the basal diet, while the treatment groups were supplemented with 120 mg/kg HF (HF120), 120 mg/kg ST (ST120), 120 mg/kg HF + 120 mg/kg ST (HF120+ST120), 120 mg/kg HF + 60 mg/kg ST (HF120+ST60), 60 mg/kg HF + 120 mg/kg ST (HF60+ST120), 60 mg/kg HF + 60 mg/kg ST (HF60+ST60). Previous studies have shown that supplementing 60 mg/kg of HF significantly improves egg production and reproductive performance in breeder hens (Dai et al., 2021). Building upon this, to ensure a balance between efficacy and safety, HF (60 mg/kg) and its doubled dose (120 mg/kg) were combined with ST in ratios of 1:1, 1:2, and 2:1 to investigate their synergistic effects. Additionally, previous studies confirmed that the maximum dose of HF and ST (120 mg/kg) used in this experiment did not exceed the safe limit, ensuring its safety for the animals (Dai et al., 2021, 2024; Jiang et al., 2020). All hens were housed in cages with ad libitum feeding and watering. The light duration of experiment house was 16 h/d. Relative humidity was maintained at 50% to70 %. Immunization and other management were performed according to the standard procedure.

### 2.4 Diet composition and nutritive values

The basal diet was formulated according to the nutritional requirements of the Feeding Standard of Chicken NY/T 33-2004 (China National Standard, 2004). Its composition and nutritional levels are shown in Table 1. The metabolizable energy (ME) and available phosphorus were calculated according to the Tables of Feed Composition and Nutritive Values (China Feed Database, 2020). The nitrogen (N) content in the feed was determined using a Kjeldahl nitrogen analyzer (KT200, FOSS Analytical A/S, Hillerød, Denmark) according to the GB/T 6432-2018 (China National Standard, 2018a), and crude protein content was calculated using formula (N × 6.25). The calcium content was determined by titration with potassium permanganate solution after treating the feed stuffs with concentrated hydrochloric acid and nitric acid, in accordance with the GB/T 6436-2018 (China National Standard, 2018b). Hydrolyzed feed samples were used to analyze lysine, methionine, and cysteine content using a fully automated amino acid analyzer (L-8900, Hitachi Ltd., Tokyo, Japan) following the methods specified in the Determination of Amino Acids in Feeds (GB/T 18246–2019, China National Standard, 2019). The crude ash content of the feed was determined according to the GB/T 6438-2007 (China National Standard, 2007), while the organic matter was calculated by subtracting the crude ash from the total feed content.

**Table 1:** Ingredient compositions and nutrient levels of basic diet (DM basis, %).

Ingredients	Content	Compositions	Content
Corn	63.70	Metabolizable energy, MJ/kg	11.67
Soybean meal	17.64	Crude protein	15.18
Corn gluten meal	2.43	Calcium	3.85
Soybean oil	1.16	Available phosphorus	0.40
Wheat bran	2.56	Lysine	0.79
Limestone	9.51	Methionine	0.39
Dicalcium phosphate	1.42	Methionine + cystine	0.65
NaCl	0.24	Organic matter	89.31
L-Lysine hydrochloride	0.15		
DL-Methionine	0.13		
Mineral prmix^1^	0.20		
Choline chloride (50%)	0.18		
Vitamin premix^2^	0.03		
Antioxidants^3^	0.03		
Phytase	0.02		
Sodium bicarbonate	0.16		
Zeolite	0.44		
Total	100.00		

1 The analytical values per kg of mineral premix composition were as follows: Cu, 8 mg; Zn, 75 mg; Fe, 80 mg; Mn, 100 mg; Se, 0.15 mg; I, 0.35 mg.

2 The analytical values per kg of vitamin premix composition were as follows: vitamin K_3_, 2.65 mg; vitamin B_1_, 2 mg; vitamin B_2_, 6 mg; vitamin B_12_, 0.025 mg; biotin, 0.0325 mg; folic acid, 1.25 mg; pantothenic acid, 12 mg; niacin, 50 mg; vitamin A, 12,500 IU; vitamin D_3_, 2500 IU; vitamin E, 30 IU.

3 The main components of antioxidants are ethoxyquin and butylated hydroxytoluene.

### 2.5 Sample collection

At the end of the 8th week of the experiment, five eggs were randomly collected from each replicate on two consecutive days (a total of 60 eggs per treatment) to determine egg quality and eggshell mechanical properties. Additionally, one eggshell sample was collected from each replicate (a total of 6 eggs per treatment) for ultrastructural observation. One chicken per replicate was selected for euthanasia, blood was collected, and serum was separated and stored at −20 °C for subsequent analysis. Microbial samples from the uterus were collected using a swab and stored in suspension in a cryovial containing 2-mL of phosphate-buffered saline (PBS; Beijing Solarbio Science Technology Co., Ltd., Beijing, China). Uterine and duodenal tissue samples were collected in appropriate amounts and stored at −80 °C, with an additional portion fixed in 4% paraformaldehyde solution (Wuhan Service Biotechnology Co., Ltd., Wuhan, Hubei, China) for subsequent sectioning.

### 2.6 Measurement of growth performance

The number and weight of both normal and abnormal eggs (broken or soft-shelled eggs) were recorded daily for each replicate throughout the experiment. Additionally, feed consumption for each replicate was recorded weekly. From week 1 to 8, the parameters were calculated for each replicate, including the average daily feed intake, average egg weight, feed-to-egg ratio, egg production rate, broken egg rate, and soft-shelled egg rate.

### 2.7 Eggshell thickness and eggshell mechanical properties

A multi-functional egg quality analyzer (DET-60000, Nanbei You Machinery Co., Ltd., Shanghai, China) was used to measure eggshell strength. The long and short diameters of the egg were measured using a digital micrometer (Mahr 40E, Nanjing SuCe Measurement Instrument Co., Ltd., Nanjing, Jiangsu, China), and the egg shape index was calculated. The eggshell thickness was measured at three points—blunt, equatorial, and sharp ends—using electronic micrometer (DL321025B, Ningbo Deli Tools Co., Ltd., Ningbo, Zhejiang, China), and the mean values were calculated. The eggshell stiffness was measured with a physical structure analyzer (TA. XTplus Texture Analyser, Stable Micro Systems, Godalming, UK) following established methods (Feng et al., 2020). The eggshell toughness was calculated using the published equation (Mabe et al., 2003).

$$\text{Fracture}\;\text{toughness}\;\left(N/{\text{mm}}^{3/2}\right)=0.777\times [2.388+(2.9934\times 6/R)]\times \left(F/T^{3/2}\right)\text{,}$$

where *F* represents eggshell breaking strength (N); *T* represents the eggshell thickness (mm); *R* is the equatorial section radius (mm).

### 2.8 Eggshell ultrastructure

The ultrastructural observation of eggshells from the CON, HF120, ST120, and HF120+ST120 groups was performed based on previously reported methods with appropriate modifications ([Bibr bib45][Bibr bib46]). After cleaning the inner and outer surfaces of the eggshells with double-distilled water to remove any contaminants, the samples were immersed in a 2% NaOH solution overnight to eliminate the shell membranes. Subsequently, the eggshells were thoroughly rinsed with water and air-dried at room temperature for 24 h. Dried samples were then cut into approximately 1-cm^2^ pieces, affixed to conductive carbon tape, and coated with gold for about 30 s using an ion sputter coater (HITACHI MC1000, Hitachi Ltd., Tokyo, Japan). The cross-sectional and surface structures of the eggshells were then examined using a scanning electron microscope (HITACHI Regulus 8100, Hitachi Ltd., Tokyo, Japan). The thicknesses of cross-sectional mammillary layer, effective layer, total thickness, and mammilla width were determined and calculated using Image-Pro Plus 6.0 software (Media Cybernetics Inc., Silver Spring, ML, USA). The mammillary layer ratio and palisade layer ratio was also calculated. Additionally, abnormal structures in the mammillary layer, including B-type mammillary, early fusion of mammillae, late fusion of mammillae, and cuff structure of mammillae, were analyzed according to previously established methods (Feng et al., 2020).

### 2.9 Calcium (Ca) and phosphorus (P) levels in the serum and uterus

Ca and P levels in serum and uterine tissues of laying hens from the CON, HF120, ST120, and HF120+ST120 groups were measured according to the method described by San et al. (2021). Ca concentration was determined using the methyl thymol blue (MTB) microplate method, and P concentration was assessed using the phosphomolybdic acid method. Both measurements were conducted with commercial kits (C004-2-1 and C006-1-1, Nanjing Jiancheng Bioengineering Institute, Nanjing, Jiangsu, China) in accordance with the manufacturer's instructions.

### 2.10 Histologic structure of the uterus and duodenum

Uterine and duodenal tissues from the CON, HF120, ST120, and HF120+ST120 groups were fixed in 4% paraformaldehyde and subsequently embedded in paraffin. The wax blocks were sectioned into 5 μm slices using a microtome (RM2016, Shanghai Leica Instrument Co., Ltd., Shanghai, China), followed by hematoxylin and eosin (H&E) staining. The uterine fold height, fold width, and epithelial height were measured using Image Pro Plus software 6.0 (Media Cybernetics Inc., Silver Spring, ML, USA). Additionally, the height of duodenal villus height (VH) and crypt depth (CD) were quantified, and the ratio of VH to CD (VH/CD) was calculated.

### 2.11 Antioxidant capacity of the uterus

The total antioxidant capacity (T-AOC) and malondialdehyde (MDA) content, as well as glutathione peroxidase (GSH-Px) activity in uterine tissues from the CON and HF120+ST120 groups were determined using commercial assay kits (A015-2-1, A003-1-2, and A005-1-2, Nanjing Jiancheng Bioengineering Institute, Nanjing, Jiangsu, China), following the operating instruction for the reagent kit.

### 2.12 Inflammatory factor levels in the uterus

The levels of interleukin (IL)-4 and IL-10 in uterine tissues from the CON and HF120+ST120 groups were measured using enzyme-linked immunosorbent assay (ELISA) kits (YJ059838 and YJ059830, Nanjing Jiancheng Biology Engineering Institute, Nanjing, Jiangsu, China), following the operating instructions.

### 2.13 Total RNA extraction, cDNA library preparation and sequencing

Total RNA was extracted from uterine and duodenal tissues of the CON and HF120+ST120 groups using the Trizol kit method (TaKaRa Bio Inc., Kyoto, Japan). The RNA concentration and quality were assessed using a Nanophotometer (NanoDrop One, Thermo Scientific Inc., Wilmington, NC, USA), while the RNA integrity was evaluated with the Agilent 2100 Bio Analyzer (Agilent Technologies Inc., Santa Clara, CA, USA). RNA samples from the uterine tissues meeting quality standards were used as starting material for mRNA library construction. Sequencing libraries were prepared and sequenced on the Illumina NovaSeq 6000 platform (Shanghai Personal Biotechnology Co., Ltd., Shanghai, China). Low-quality sequences (<20 nucleotide) and adapter sequences were removed to obtain high-quality reads. The filtered reads were then aligned to the chicken reference genome (Gallus_gallus.GRCg7b) using the HISAT2 tool. Gene expression levels were quantified using the Fragments Per Kilobase of Transcript per Million Fragments Mapped (FPKM) method. Differential expression analysis between samples was conducted using the DESeq2 package, with the Benjamini-Hochberg method applied to control the false discovery rate (FDR) and adjust *P*-value. A fold change |fold change| > 1.5 at *P* < 0.05 was used as the criterion for identifying differentially expressed genes (DEGs) between groups. Functional annotation of DEGs was conducted using Gene Ontology (GO) and Kyoto Encyclopedia of Genes and Genomes (KEGG) enrichment analyses, with *P* < 0.05 as the threshold for significant enrichment.

### 2.14 Real-time quantitative PCR

Total RNA was reverse-transcribed into cDNA using the PrimeScript RT kit (TaKaRa Biotechnology Co., Ltd., Dalian, Liaoning, China). The mRNA expression levels of genes related to the duodenum, transcriptome sequencing validation, and other associated genes were quantified using a SYBR Premix Ex Taq II kit (Takara Biomedical Technology Co., Ltd., Beijing, China) on a 7500 fluorescence detection system (Applied Biosystems LLC., Waltham, MA, USA). Gene-specific primer sequences are provided in Table S1. Relative gene expression levels were calculated using the 2^−ΔΔCt^ method, with β-actin serving as the internal reference gene.

### 2.15 The rRNA sequencing and data analysis of microorganisms in the uterus

Microbial DNA from the uterine tissues was extracted from 12 samples using the OMEGA Soil DNA kit (M5635–02, OMEGA Bio-Tek, Norcross, GA, USA). The V3–V4 region of the 16S rRNA gene was amplified using primer pairs 338F (5′-ACTCCTACGGGGAGGCAG-3′) and 806R (5′-GGACTACHVGGGTW TCTAAT-3′). The abundance and quality of the amplicon libraries were assessed using an Agilent 2100 Bioanalyzer (AgilentTechnologies Inc., Santa Clara, CA, USA) and a Library Quantification kit for Illumina (KK4824, Kapa Biosciences, Woburn, MA, USA), respectively. 16S rRNA libraries were sequenced on the Illumina NovaSeq 6000 SP platform (LC Biotechnology Co., Ltd, Shanghai, China). Sequences were compared with the Greengenes database (silva_138_1). Sequence data analyses were primarily performed using the QIIME2 and R software packages (v3.2.0). Alpha diversity was analyzed to evaluate the complexity of species diversity, and β diversity was calculated using QIIME2. Linear discriminant analysis (LDA) combined with effect size measurement (LefSe) was applied to determine the relative abundance of taxa between groups (LDA > 4, *P* < 0.05).

### 2.16 Statistical analysis

One-way ANOVA was performed using SPSS software (version 21.0, SPSS Inc., Chicago, IL, USA). The statistical model used was as follows:

$$Y_{ij}=\mu +\alpha_{i}+\varepsilon_{ij}\text{,}$$

where *Y*_*ij*_ is the observation of dependent variables, *μ* is the overall mean, *α*_*i*_ is the fixed effect of treatment, and *ε*_*ij*_ is the random error for the observation. Duncan's method was used for multiple comparisons when data differences were significant. Differences between the two groups were analyzed using the independent sample *t*-test procedure. Wilcoxon rank-sum tests were employed to examine differences in the relative abundance of uterine microorganisms between the two groups. Spearman's correlation test was applied to explore relationships among multiple variables. Statistical significance was defined as *P* < 0.05 for a significant difference, *P* < 0.01 for an extremely significant difference.

## 3 Results

### 3.1 Production performance

**Fig. 1:**
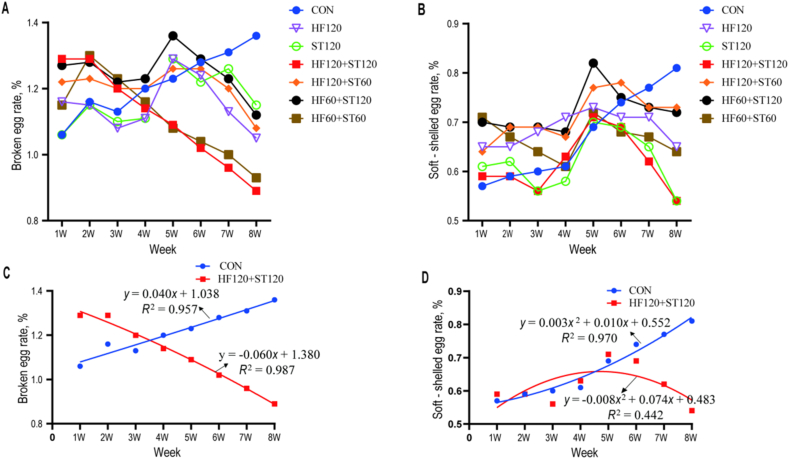
Effect of dietary supplementation with HF and ST on the broken egg rate and soft-shelled egg rate (*n* = 6). (A) and (B) represent the egg production rate, broken egg rate, and soft-shelled eggs of laying hens from 1 to 8 week, respectively. (C) and (D) show the regression fitting curves and equations of broken egg rate and soft-shelled egg rate with experimental weeks of age for the control group and HF120+ST120 group, respectively. CON, basal diet; HF120, basal diet supplemented with 120 mg/kg HF; ST120, basal diet supplemented with 120 mg/kg ST; HF120+ST120, basal diet supplemented with 120 mg/kg HF and 120 mg/kg ST; HF120+ST60, basal diet supplemented with 120 mg/kg HF and 60 mg/kg ST; HF60+ST120, basal diet supplemented with 60 mg/kg HF and 120 mg/kg ST; HF60+ST60, basal diet supplemented with 60 mg/kg HF and 60 mg/kg ST. HF = hawthorn leaf flavonoids; ST = stevioside.

As shown in Table 2, no significant differences (*P* > 0.05) were observed among the treatment groups and the CON group in the egg laying rate, average egg weight, daily feed intake, and FCR during week 1 to 8. As shown in Fig. 1. A and B, statistical analysis was performed on the rates of weekly broken egg and soft-shelled egg during the experimental period, which could display the dynamic trends of various indexes with the treatment time of additives. Compared with the CON group, Although there was no significant difference in the broken egg rate and soft-shelled egg rate among all groups, the treatment groups showed a numerical decrease at the 8th week of the experiment. Moreover, it was found that the HF120+ST120 group had the best effect in reducing the incidence of broken eggs and soft-shelled eggs, with a numerical decrease of 0.47% and 0.27% respectively. Therefore, the data of the CON group and HF120+ST120 group were selected to plot fitting curves for further analysis (Fig. 1C and D). The broken egg rate of the CON group showed a linear increasing trend with the increase of time, while that of the HF120+ST120 group showed a linear decreasing trend. The soft-shelled egg rate of the CON group showed a quadratic curve increasing trend with the increase of time, while that of the HF120+ST120 group increased gradually in the first 4 weeks and then showed a quadratic curve decreasing trend.

**Table 2:** Effects of dietary supplementation of HF and ST on the egg laying performance.

Item^1^	Egg laying rate, %	Average egg weight, g	ADFI, g/d	FCR
CON	81.79	57.53	107.08	2.34
HF120	79.95	57.98	103.75	2.35
ST120	80.32	57.57	102.28	2.38
HF120+ST120	80.07	58.18	103.13	2.29
HF120+ST60	80.33	58.38	107.01	2.39
HF60+ST120	79.90	58.37	106.37	2.34
HF60+ST60	80.59	58.13	101.73	2.33
SEM	2.075	0.165	0.848	0.024
*P*-value	0.997	0.727	0.410	0.966

HF = hawthorn leaf flavonoids; ST = stevioside; ADFI = average daily feed intake; FCR = feed conversion ratio; SEM = standard error of the mean.

1 CON, basal diet; HF120, basal diet supplemented with 120 mg/kg HF; ST120, basal diet supplemented with 120 mg/kg ST; HF120+ST120, basal diet supplemented with 120 mg/kg HF, and 120 mg/kg ST; HF120+ST60, basal diet supplemented with 120 mg/kg HF, and 60 mg/kg ST; HF60+ST120, basal diet supplemented with 60 mg/kg HF, and 120 mg/kg ST; HF60+ST60, basal diet supplemented with 60 mg/kg HF, and 60 mg/kg ST; *n* = 6.

### 3.2 Eggshell thickness and mechanical properties

As shown in Table 3, The breaking strength was significantly higher in the HF120, HF120+ST120, HF120+ST60, and HF60+ST60 groups compared to the CON group (*P* = 0.003). No significant differences in the egg shape index (*P* = 0.375) and eggshell stiffness (*P* = 0.276) were observed among the treatment groups and the CON group. However, the eggshell thickness was significantly greater in the HF120+ST120 group compared with the CON group (*P* = 0.011). Moreover, compared to the CON group, the eggshell fracture toughness was significantly higher in both the HF120+ST120 and HF120+ST60 groups (*P* = 0.048). Especially, the HF120+ST120 treatment exhibited the best effect on enhancing the eggshell fracture toughness.

**Table 3:** Effects of dietary supplementation with HF and ST on the eggshell thickness and mechanical properties of laying hens.

Item^1^	Shape index	Breaking strength, N	Shell thickness, mm	Shell stiffness, N/mm	Fracture toughness, N/mm^3/2^
CON	1.32	3.31^c^	0.330^bc^	136.27	433.68^c^
HF120	1.31	3.63^ab^	0.338^abc^	139.62	454.40^abc^
ST120	1.31	3.60^bc^	0.335^bc^	138.43	443.63^bc^
HF120+ST120	1.31	3.94^a^	0.350^a^	148.53	487.01^a^
HF120+ST60	1.32	3.84^ab^	0.342^ab^	139.08	470.81^ab^
HF60+ST120	1.35	3.52^bc^	0.327^c^	136.36	455.87^abc^
HF60+ST60	1.31	3.71^ab^	0.343^ab^	139.76	461.74^abc^
SEM	0.009	0.046	0.0024	1.396	4.688
*P*-value	0.375	0.003	0.011	0.276	0.048

HF = hawthorn leaf flavonoids; ST = stevioside; SEM = standard error of the mean.

Within a column, means without a common superscript letter differ at *P* < 0.05 (*n* = 6).

1 CON, basal diet; HF120, basal diet supplemented with 120 mg/kg HF; ST120, basal diet supplemented with 120 mg/kg ST; HF120+ST120, basal diet supplemented with 120 mg/kg HF, and 120 mg/kg ST; HF120+ST60, basal diet supplemented with 120 mg/kg HF, and 60 mg/kg ST; HF60+ST120, basal diet supplemented with 60 mg/kg HF, and 120 mg/kg ST; HF60+ST60, basal diet supplemented with 60 mg/kg HF, and 60 mg/kg ST.

### 3.3 Eggshell ultrastructure

**Fig. 2:**
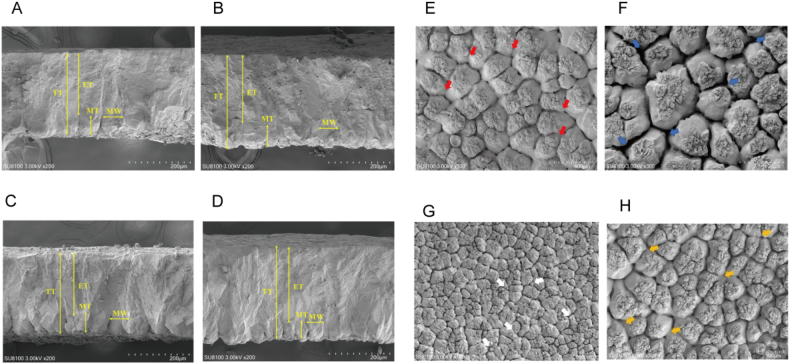
The ultrastructure observation of eggshells. (A), (B), (C), and (D) show images of the vertical surfaces of the eggshell ultrastructure in the CON, HF120, ST120, and HF120+ST120 groups, respectively. (E), (F), (G), and (H) display the structure of the mammillary layer of the eggshell, with red arrows indicating early fusion, blue arrows indicating late fusion, white arrows indicating B-model mammillary structures, and yellow arrows highlighting cuff structures. CON, basal diet; HF120, basal diet supplemented with 120 mg/kg HF; ST120, basal diet supplemented with 120 mg/kg ST; HF120+ST120, basal diet supplemented with 120 mg/kg HF and 120 mg/kg ST. HF = hawthorn leaf flavonoids; ST = stevioside; TT = total thickness; ET = effective layer thickness; MT = mammillary layer thickness; MW = mammillary layer width.

As shown in Table 4 and Fig. 2, compared to the CON group, the HF120+ST120 group exhibited significantly increased the thickness and proportion of the eggshell effective layer (*P* < 0.05), while the proportion of mammillary layer was significantly reduced (*P* = 0.045). There were no significant differences in the thickness and width of the mammillae among all groups. However, the total thickness of the eggshell ultrastructure was significantly greater in the HF120, ST120 and HF120+ST120 groups compared with the CON group (*P* = 0.003). Compared to the CON group, the frequency of early fusion in mammillae was significantly higher in both the ST120 and HF120+ST120 groups (*P* = 0.001), with the HF120+ST120 group showing a significantly greater early fusion frequency than the ST120 group (*P* < 0.05). No significant differences were observed in the late fusion and cuff structure among all groups, but the number of B-type mammillae was significantly reduced in the HF120+ST120 group compared with the CON group (*P* = 0.034).

**Table 4:** Effects of dietary supplementation with HF and ST on the eggshell ultrastructure of laying hens.

Item^1^	CON	HF120	ST120	HF120+ST120	SEM	*P*-value
Mammillary thickness, mm	68.87	75.05	74.80	74.89	1.208	0.198
Effective layer thickness, mm	188.68^c^	213.85^bc^	221.25^ab^	244.54^a^	5.833	0.002
Total thickness, mm	261.09^b^	292.58^a^	299.86^a^	323.25^a^	6.627	0.003
Mammillary layer width, mm	66.90	61.68	66.81	65.46	1.069	0.280
Mammillary layer ratio, %	26.59^a^	25.67^ab^	25.06^ab^	23.18^b^	0.456	0.045
Effective layer ratio, %	72.10^b^	73.09^b^	73.67^ab^	75.64^a^	0.454	0.031
Early fusion 2	1.72^c^	1.96^bc^	2.45^b^	3.17^a^	0.156	0.001
Late fusion 2	3.78	3.20	2.95	2.50	0.184	0.088
Type B mammillae 2	4.00^a^	2.49^ab^	2.58^ab^	1.75^b^	0.289	0.034
Cuff-like structure 2	0.95	0.88	1.11	1.06	0.103	0.868

HF = hawthorn leaf flavonoids; ST = stevioside; SEM = standard error of the mean.

Within a row, means without a common superscript letter differ at *P* < 0.05 (*n* = 6).

1 CON, basal diet; HF120, basal diet supplemented with 120 mg/kg HF; ST120, basal diet supplemented with 120 mg/kg ST; HF120+ST120, basal diet supplemented with 120 mg/kg HF, and 120 mg/kg ST.

2 The number of typical structures of a unit area appear in random sample photography.

### 3.4 Serum and uterine Ca and P levels

As shown in Table 5, there were no significant differences in the content of P in the serum and the levels of Ca and P in the uterus among all groups. However, compared to the CON group, the serum Ca level was significantly increased in the HF120+ST120 group (*P* = 0.020).

**Table 5:** Effects of dietary supplementation with HF and ST on the calcium and phosphorus levels in the serum and uterus of laying hens (mol/L).

Item^1^	CON	HF120	ST120	HF120+ST120	SEM	*P*-value
**Serum**						
Ca	1.57^b^	1.60^b^	1.88^ab^	2.08^a^	0.072	0.020
P	0.78	0.61	0.82	0.72	0.036	0.197
**Uterus**						
Ca	0.21	0.16	0.15	0.17	0.009	0.151
P	1.70	1.51	1.62	1.81	0.068	0.490

HF = hawthorn leaf flavonoids; ST = stevioside; SEM = standard error of the mean.

Within a row, means without a common superscript letter differ at *P* < 0.05 (*n* = 6).

1 CON, basal diet; HF120, basal diet supplemented with 120 mg/kg HF; ST120, basal diet supplemented with 120 mg/kg ST; HF120+ST120, basal diet supplemented with 120 mg/kg HF, and 120 mg/kg ST.

### 3.5 Morphological structure of duodenal and uterine tissues

**Fig. 3:**
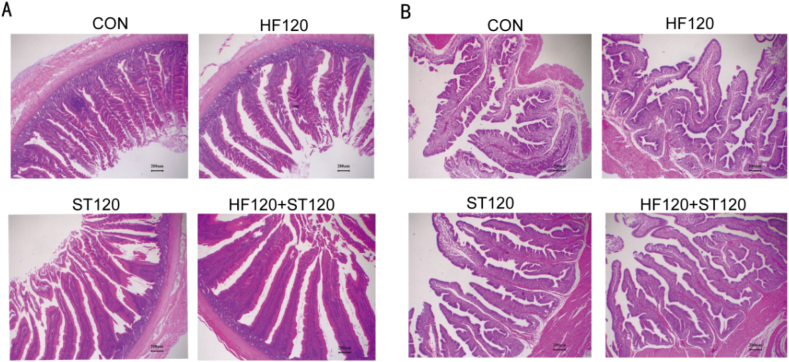
The histological observation of the duodenum and uterus of laying hens. (A) Histological observation of duodenum. (B) Histological observation of the uterine villi folds. CON, basal diet; HF120, basal diet supplemented with 120 mg/kg HF; ST120, basal diet supplemented with 120 mg/kg ST; HF120+ST120, basal diet supplemented with 120 mg/kg HF and 120 mg/kg ST. Magnification 40×, scale bar 200 μm. HF = hawthorn leaf flavonoids; ST = stevioside.

As shown in Table 6 and Fig. 3, compared to the CON group, there were no significant differences in CD among the treatment groups. However, duodenal VH and the VH/CD were significantly higher in the HF120, ST120, and HF120+ST120 groups (*P* < 0.05). Additionally, the VH/CD in the HF120+ST120 group was significantly higher than that in the HF120 and ST120 groups (*P* < 0.001). Compared to the CON group, there were no significant differences in the width of uterine villi folds and epithelial height in all treatment groups of laying hens. However, the height of uterine villi folds was significantly increased in the ST120 and HF120+ST120 groups (*P* = 0.001).

**Table 6:** Effects of dietary supplementation with HF and ST on the histomorphology of the duodenum and uterus of laying hens (μm).

Item^1^	CON	HF120	ST120	HF120+ST120	SEM	*P*-value
**Duodenum**						
VH	1340^b^	1603^a^	1839^a^	1849^a^	59.5	0.001
CD	267.3	241.9	243.4	218.2	7.63	0.157
VH/VD	5.17^c^	6.67^b^	7.64^ab^	8.54^a^	0.340	<0.001
**Uterus**						
Height of mucosal villus	1678^c^	2010^bc^	2306^ab^	2703^a^	103.7	0.001
Width of mucosal villus	385.4	325.4	364.9	392.5	12.02	0.196
Epithelial height	38.61	39.42	42.39	44.94	1.001	0.088

HF = hawthorn leaf flavonoids; ST = stevioside; VH = villus height; CD = crypt depth; VH/VD = the ratio of VH to CD; SEM = standard error of the mean.

Within a row, means without a common superscript letter differ at *P* < 0.05 (*n* = 6).

^1^CON, basal diet; HF120, basal diet supplemented with 120 mg/kg HF; ST120, basal diet supplemented with 120 mg/kg ST; HF120+ST120, basal diet supplemented with 120 mg/kg HF, and 120 mg/kg ST.

### 3.6 Relative mRNA expression of duodenal Ca transporter genes

**Fig. 4:**
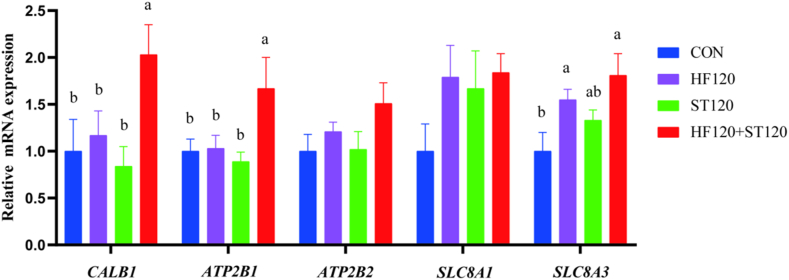
Effects of dietary supplementation with HF and ST on the relative mRNA expression of duodenal calcium transporter genes in laying hens. CON, basal diet; HF120, basal diet supplemented with 120 mg/kg HF; ST120, basal diet supplemented with 120 mg/kg ST; HF120+ST120, basal diet supplemented with 120 mg/kg HF and 120 mg/kg ST. Lower case letters in the bar chart indicate significant differences (*P* < 0.05). HF = hawthorn leaf flavonoids; ST = stevioside. Different lowercase letters above columns represent significant differences among treatments at *P* < 0.05 (*n* = 6).

As shown in Fig. 4, the relative expression levels of mRNAs, including duodenal *CALB1* and *ATP2B1*, were significantly increased in the HF120+ST120 group compared to the CON group (*P* < 0.05). The relative expression of duodenal *SLC8A3* mRNA was also significantly higher in the HF120 and HF120+ST120 groups compared to the CON group (*P* = 0.022).

### 3.7 Identification of DEGs in the uterus

**Fig. 5:**
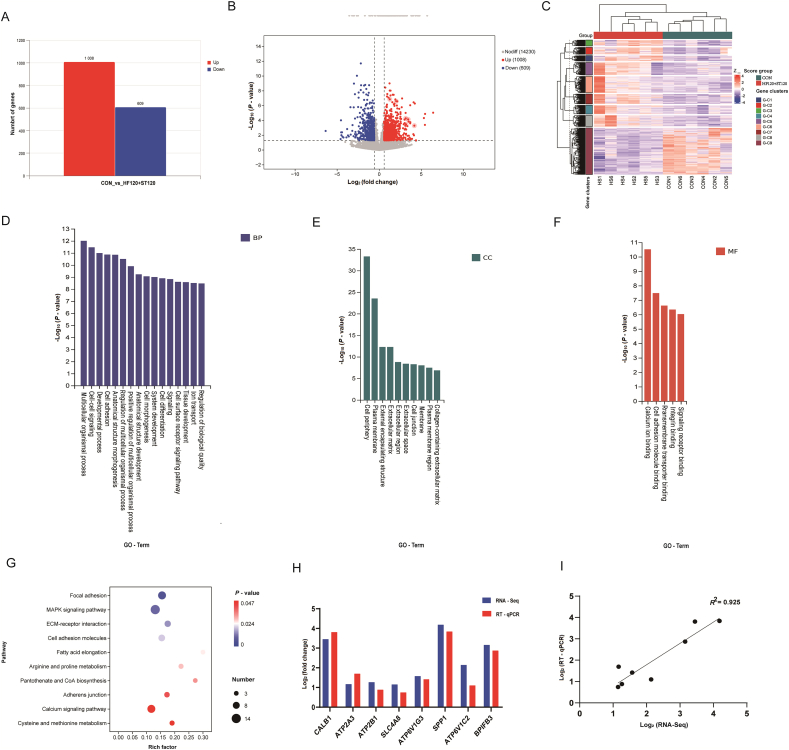
RNA-Seq analysis of the uterus and validation in laying hens of the CON and HF120+ST120 groups. (A) The differentially expressed genes (DEGs; |fold change| > 1.5 and *P* < 0.05. (B) Volcano diagram of DEGs. (C) Hierarchical clustering analysis of DEGs in the uterus. (D) GO analysis of DEGs in the uterus (biological process, BP). (E) GO analysis of DEGs in the uterus (cellular component, CC). (F) GO analysis of DEGs in the uterus (molecular function, MF). (G) KEGG pathway analysis of DGEs in the uterus. (H) RT-qPCR validation of RNA-Seq accuracy. (I) RNA-Seq and RT-qPCR correlation analysis. CON, basal diet; HF120+ST120, basal diet supplemented with 120 mg/kg HF and 120 mg/kg ST; *n* = 6. HF = hawthorn leaf flavonoids; ST = stevioside; GO = Gene Ontology; KEGG = Kyoto Encyclopedia of Genes and Genomes; RNA-Seq = RNA sequencing.

As shown in Table S2, the 12 libraries constructed from uterine tissue samples of the two treatment groups yielded a total of 625,029,884 clean reads, with more than 36,929,210 clean reads per sample. Between 90.70% and 93.44% of the reads were mapped to the reference genome. The GC content ranged from 49.41% to 52.01%, and over 95.19% of the bases had a quality score of ≥Q30. Compared to the CON group, a total of 1617 DEGs were identified in the HF120+ST120 group, with 1008 upregulated and 609 down-regulated genes (Fig. 5A). Additionally, intergroup DGEs were visualized in a volcano plot (Fig. 5B). Hierarchical clustering analysis of the samples in each group revealed that the CON group and HF120+ST120 group clustered separately, demonstrating the accuracy of the samples from our bioinformatics analysis and the reliability of the DEGs (Fig. 5C).

### 3.8 Gene Ontology and KEGG analysis of DEGs

The differential gene GO-enriched biological processes were primarily associated with processes related to normal chicken growth and development, as well as mineralized ion transport. These processes included the regulation of biological quality, ion transport, tissue development, cell surface receptor signaling, cell differentiation, cell morphogenesis, positive regulation of multicellular organismal processes, cell adhesion, cell–cell signaling, and multicellular organismal processes (Fig. 5D). Cellular components identified included the extracellular region, extracellular matrix, plasma membrane region, and collagen-containing extracellular matrix (Fig. 5E). Molecular functions encompassed signaling receptor binding, transmembrane transporter binding, cell adhesion molecule binding, and calcium ion binding (Fig. 5F). Additionally, KEGG pathway analysis of upregulated DEGs revealed the top 10 significantly enriched pathways, as shown in Fig. 5G. Among these pathways, the highest number of differentially enriched genes were associated with the MAPK signaling pathway, the adhesion patch, and the calcium signaling pathway. Based on the gene expression trends from the enriched pathways, 13 transporter carrier genes associated with eggshell mineralization (*CALB1*, *NKAIN4*, *ATP6V0D2*, *ATP6V1C2, ATP6V1G3*, *ATP2B1*, *ATP13A5*, *ATP2A3*, *CA4*, *SLC4A8*, *KCNH1*, *KCNMA1*, and *ANXA5*) and 5 genes encoding matrix proteins (*GPC4*, *MEPE*, *BPIFB3*, *SPP1*, and *CLU*) were identified.

### 3.9 Quantitative real-time PCR (qRT-PCR) validation of RNA-Seq data

As shown in Fig. 5H I, eight genes were selected for qRT-PCR validation, and the mRNA expression levels obtained from qRT-PCR were consistent with those from transcriptome analysis. The RNA-Seq and qRT-PCR data were highly correlated, with a correlation coefficient of 0.925, indicating the reliability of the RNA-Seq data.

### 3.10 Antioxidant capacity and anti-inflammatory factor content in the uterus

As shown in Table 7, T-AOC and GSH-Px activities were significantly higher (*P* < 0.05), while MDA levels were significantly lower in the HF120+ST120 group compared with the CON group (*P* = 0.013). The levels of uterine IL-4 and IL-10 were significantly higher in the HF120+ST120 group compared to the CON group (*P* < 0.05).

**Table 7:** Effects of dietary supplementation with HF and ST on the antioxidant capacity and inflammatory factor content in the uterus of laying hens.

Item^1^	CON	HF120+ST120	SEM	*P*-value
T-AOC, U/g prot	29.38^b^	42.33^a^	3.128	0.030
MDA, nmol/mg prot	1.89^a^	1.25^b^	0.141	0.013
GSH-Px, U/mg prot	51.84^b^	69.44^a^	3.766	0.011
IL-4, pg/mg prot	107.1^b^	119.8^a^	3.26	0.044
IL-10, pg/mg prot	186.9^b^	236.2^a^	11.46	0.022

HF = hawthorn leaf flavonoids; ST = stevioside; SEM = standard error of the mean; prot = protein.

Within a row, means without a common superscript letter differ at *P* < 0.05 (*n* = 6).

1 CON, basal diet; HF120+ST120, basal diet supplemented with 120 mg/kg HF, and 120 mg/kg ST.

### 3.11 Spearman's correlation analysis of RNA-Seq differential genes and key indicators with significant difference

**Fig. 6:**
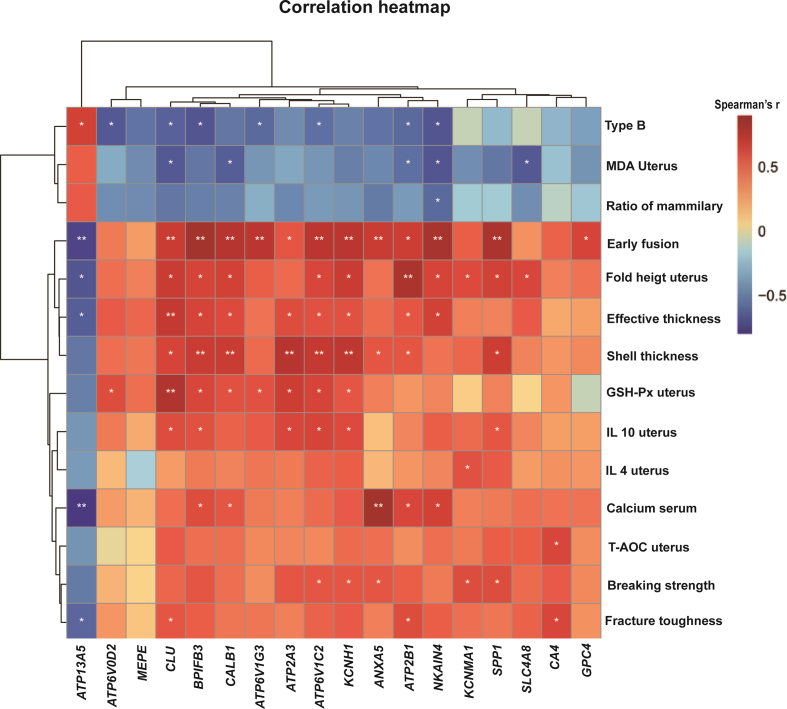
Heatmap of Spearman's correlation coefficients between transcriptome screening DEGs and key indicators of significant difference. The red and blue color represent positive and negative correlation, respectively. ∗ indicates *P* < 0.05, ∗∗ indicates *P* < 0.01. MDA = malondialdehyde; GSH-Px = glutathione peroxidase; IL = interleukin; T-AOC = total antioxidant capacity.

As shown in Fig. 6, Spearman's correlation analysis were conducted to investigate the relationship between uterine health, eggshell phenotypic indicators, and DEGs identified through transcriptome sequencing in laying hens. The results indicated that the transcription levels of *CALB1*, *ATP2B1*, *CLU*, *BPIFB3*, *ATP6V1C*2, and *KCNH1* were positively correlated with the frequency of early fusion in egg shell mammillae, eggshell thickness, effective layer thickness, and uterine villi fold height (*P* < 0.05). Additionally, the transcription levels of *ATP2B1*, *CLU*, *BPIFB3*, *ATP6V1G3*, *ATP6V1C2*, *ATP6V0D2*, and *NKA1N4* were negatively correlated with the number of B-type mammillae (*P* < 0.05). The transcription levels of *SPP1*, *KCNH1*, *KCMA1*, *ATP6V1C2*, and *ANXA5* were positively correlated with the breaking strength (*P* < 0.05). Furthermore, the transcription levels of *CALB1*, *CLU*, *ATP2B1*, *NKA1N4*, and *SLC4A8* were negatively correlated with MDA content in uterine tissues (*P* < 0.05). Finally, the transcription levels of *CALB1*, *CLU*, *BPIFB3*, *ATP6V1C2*, *ATP2A3*, *ATP6V1G3*, and *KCNH1* were negatively correlated with GSH-Px activity in uterine tissues (*P* < 0.05), while *CLU*, *BPIFB3*, *ATP6V1C2*, *SPP1*, *ATP2A3*, and *KCNH1* were positively correlated with the IL-10 level (*P* < 0.05).

### 3.12 The rRNA sequencing in the uterus

**Fig. 7:**
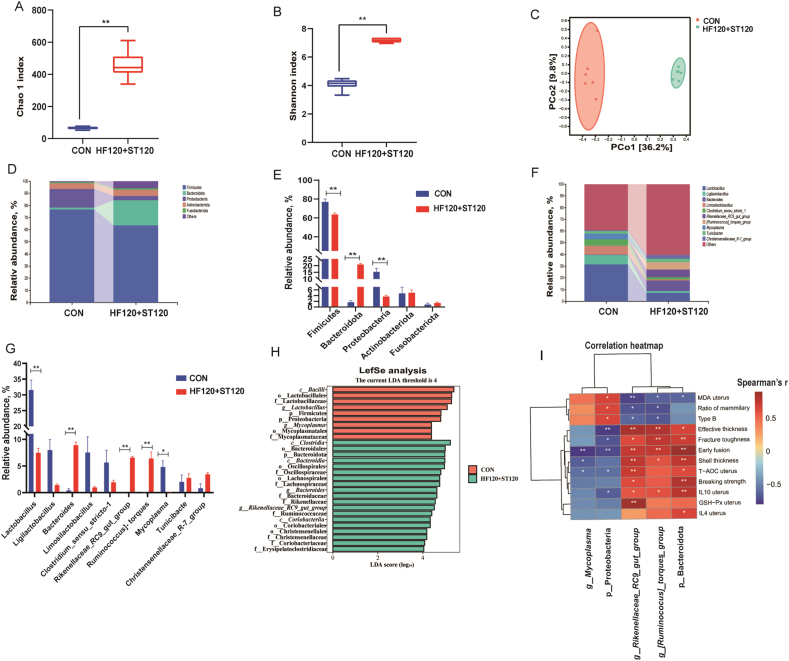
Effect of dietary supplementation with HF and ST on the bacterial microbiota in the uteru of laying hens. (A) Alpha diversity of uterus microorganisms-Chao 1. (B) Alpha diversity of uterine microorganisms-Shannon. (C) Principal coordinate analysis (PCoA). (D) Microbial composition at the phylum level. (E) Analysis of microbial diversity at the phylum level. (F) Microbial composition at the genus level. (G) Analysis of microbial diversity at the genus level. (H) LefSe analysis (LDA score 4.0). (I) Correlation analysis heatmap. CON, basal diet; HF120+ST120, basal diet supplemented with 120 mg/kg HF and 120 mg/kg ST. HF = hawthorn leaf flavonoids; ST = stevioside; LDA = linear discriminant analysis; LefSe = linear discriminant analysis effect size. ∗ indicates *P* < 0.05, ∗∗ indicates *P* < 0.01.

The results of microbial 16S rRNA sequencing in the uterus are presented in Fig. 7. Compared to the CON group, both the Chao 1 and Shannon indices were significantly higher in the HF120+ST120 group (Fig. 7A and B, *P* < 0.01). Principal coordinate analysis (PCoA) revealed a clear separation of communities between the groups, with 36.2% and 9.8% variation explained respectively by the first two principal components (PCoA1 and PCoA2), indicating a significant increase in the β-diversity in the HF120+ST120 group (Fig. 7C, *P* < 0.05). At the phylum level, the top five relative abundances of uterine microorganisms were Firmicutes, Bacteroidetes, Proteobacteria, Actinobacteria, and Fusobacteria (Fig. 7D). Compared to the CON group, the abundance of Firmicutes and Proteobacteria was significantly lower (Fig. 7E, *P* < 0.01), while the abundance of the Bacteroidetes was significantly higher in the HF120+ST120 group (*P* < 0.01). The top 10 microbiota ranked at genus level are shown in Fig. 7F. At the genus level, compared to the CON group (Fig. 7G), the abundance of *Lactobacillus* and *Mycoplasma* was significantly lower in the HF120+ST120 group (*P* < 0.05), while the abundance of Bacteroides, *Rikenellaceae_RC9_gut_group*, and *Ruminococcus* was significantly higher (*P* < 0.01). linear discriminant analysis effect size (LefSe) analysis (Fig. 7H) identified *Mycoplasma* as the dominant genera in the CON group, and *Rikenellaceae_RC9_gut_group* and Bacteroides as the dominant genera in the HF120+ST120 group.

### 3.13 Spearman's correlation analysis of differential bacterial microbiota and key indicators with significant difference in the uterus

As shown in Fig. 7I, Spearman's correlation analysis revealed that the abundance of Bacteroidetes, *Ruminococcus*, and *Rikenellaceae_RC9_gut_group* was positively correlated with eggshell thickness, effective layer thickness, frequency of early fusion in mammillae, and eggshell fracture toughness (*P* < 0.05). In contrast, the abundance of Proteobacteria was negatively correlated with eggshell effective layer thickness, eggshell fracture toughness, and frequency of early fusion in mammillae (*P* < 0.05), but positively correlated with the proportion of mammillary layer and the number of B-type mammilla (*P* < 0.05). The abundance of *Ruminococcus* and *Rikenellaceae_RC9_gut_group* was negatively correlated with the proportion of mammillary layer and the number of B-type mammilla (*P* < 0.05). Furthermore, the abundance of Bacteroidetes, *Ruminococcus*, and *Rikenellaceae_RC9_gut_group* was positively correlated with IL-10 levels in uterine tissues and negatively correlated with MDA content (*P* < 0.05). Additionally, the abundance of *Rikenellaceae_RC9_gut_group* and Bacteroidota was positively correlated with uterine tissue T-AOC, while the abundance of *Mycoplasma* and Proteobacteria was negatively correlated with uterine tissue T-AOC (*P* < 0.05). Finally, the abundance of the Bacteroidetes was positively correlated with IL-4 levels in uterine tissues, while the abundance of Proteobacteria was negatively correlated with IL-10 levels (*P* < 0.05).

## 4 Discussion

As hens approach the later stages of laying period, both egg production rate and eggshell quality decline rapidly. Some phytoflavonoids have the potential as functional feed additives by enhancing laying performance and improving economic efficiency (Liu et al., 2013). Dietary genistein supplementation has been found to improve the egg production and eggshell quality of aged laying hens (Lv et al., 2019). In the present study, the HF and ST treatments had no significant effects on the egg laying performance. However, the reduction in the weekly broken and soft-shelled egg rates was reversed gradually by the HF and ST treatments, particularly at the second half of the experimental phase by the HF120+ST120 treatment. Previous study indicated that feeding 45-week-old laying hens with phytoflavonoid genistein for 10 weeks could improve both the egg production and feed conversion (Hou et al., 2024). The discrepancies between the current results and previous studies may be attributed to the hen age and the duration of additive supplementation. Therefore, supplementing HF and ST in the diets can improve eggshell quality of laying hens, and a prolonged action time is likely to yield better results.

As the laying cycle of hens extends, the eggshell quality, particularly eggshell strength, declines significantly (Liu et al., 2013). The material and structural characteristics are important factors determining the mechanical properties of eggshells. The material properties depend on the inorganic and organic components of the eggshell and their interactions, while the structural properties depend on the thickness, density, shape, and size of the eggshell (Bain, 2005). In this study, the addition of HF and ST significantly increased the eggshell strength, with the HF120+ST120 group also showing a significant increase in the eggshell thickness. A previous study demonstrated the inclusion of mulberry leaf flavonoids could improve both eggshell thickness and strength, which aligns with the current findings (Hang et al., 2022). The results of this study revealed that both the HF120+ST120 and HF120+ST60 groups significantly enhanced eggshell fracture toughness. The HF120+ST120 group showed a more pronounced effect. Fracture toughness is a crucial indicator of eggshell resistance to external forces, and a strong positive correlation exists between eggshell strength and thickness (Fathi et al., 2019). Ultrastructural characteristics exert an important influence on the mechanical properties of eggshells (Ahmed et al., 2005). The effective layer of the eggshell, which is the primary stress-bearing layer to determining eggshell thickness and strength (Guru and Dash, 2014). In this study, dietary supplementation with HF and ST significantly increased total eggshell thickness, along with the increased thickness and proportion of the effective layer. The mammillary layer is essential for eggshell formation, and its early structural development improves the adhesion between the papillary shell membrane and promotes eggshell formation (Feng et al., 2020). The early fusion of mammillae is indicative of eggshell “quality optimization” (characterized by structural compactness and stable performance), whereas late fusion reflects mineralization abnormalities, which impair mechanical and functional integrity of the eggshell. Type B mammillae are small, abnormal round mammillae that lie free between normal ones. When external force compresses the eggshell, cracks propagate along the gaps between Type B and normal mammillae accelerate the propagation of these cracks (Bain, 1992). In the current study, dietary supplementation with HF and ST increased the frequency of early mammillary fusion, while decreasing the frequency of late fusion and B type mammillae. including increased proportion of effective layers and reduced abnormalities in mammilla structures. Greater effective layer thickness, smaller mammilla structures, and thicker palisade layers contribute to stronger eggshells (Li et al., 2018). Therefore, dietary HF and ST supplementation can enhance the eggshell quality by improving both the ultrastructure and mechanical properties.

Eggshell ultrastructure is formed through the successive precipitation of mineral carbonates and organic substrates during the mineralization phase (Jonchère et al., 2012). Ca and P are essential nutrients for this process, and an imbalanced Ca/P ratio or metabolic disturbance can impair eggshell quality (San et al., 2021). The Ca^2+^ required for eggshell formation are absorbed via transcellular pathways in the duodenal epithelium, where they enter epithelial cells through the TRPV6 calcium channel and bind to calcium-binding proteins (Fleet et al., 2010; Wongdee et al., 2015). Subsequently, Ca^2+^ is transported into the bloodstream by the plasma membrane calcium ATPase (encoding genes include *PMCA*, *ATP2B1* and *ATP2B2*) and sodium–calcium exchanger (encoding genes include *NCX SLC8A1*, and *SLC8A3*), ultimately reaching the uterus where it contributes to eggshell formation (Nie et al., 2020). However, as the laying cycle progresses, structural changes in tissues may occur, leading to functional impairment in various organs (Chen et al., 2021; Sun et al., 2023). In the present study, dietary supplementation with HF and ST for late-laying hens significantly improved the morphology of the duodenum and uterus, and upregulated the mRNA expression of key calcium transporters such as *CALB1*, *ATP2B1*, and *SLC8A3* in the duodenum. Well-maintained morphological structure of tissues and organs is essential for effective Ca transport and deposition.

With advancements in transcriptomics technology, an increasing number of genes associated with biomineralization have been identified. Eggshell formation is governed by the interaction between organic substrates and inorganic solutes, with precursors secreted by genetically regulated uterine epithelial cells (Khan et al., 2019). Ca is the most critical mineral element in eggshell mineralization, directly influencing egg quality (Fu et al., 2024). In the present study, RNA-Seq of uterine tissues identified six differentially upregulated genes (*ATP2B1*, *ATP2A3*, *ATP13A5*, *CALB1*, *ANXA5*, and *CA4*) that are directly involved in the formation and transport of Ca^2+^ and bicarbonate ion (HCO_3_^-^). CALB1, a calcium-binding protein, facilitates the transport of Ca^2+^ within uterine epithelial cells. ATP2B1, a calcium pump, transfers Ca^2+^ from uterine epithelial cells into uterine fluid to provide raw materials for eggshell formation (Nii et al., 2018). Excess calcium in the endoplasmic reticulum of uterine epithelial cells is stored via sarcoplasmic/endoplasmic reticulum Ca^2+^-ATPase, which is encoded by *ATP13A5* and *ATP2A3* (Vermassen et al., 2004). Additionally, HCO_3_^-^ in uterine fluid is partly transported by solute carrier family proteins and partly synthesized from carbon dioxide and water, catalyzed by carbonic anhydrase 4 (CA4), before being transported by SLC4A8 (Sahin et al., 2018). In addition to Ca^2+^ and HCO_3_^-^, eggshell formation involves the transport of additional ions—such as Na^+^, H^+^, and K^+^—to maintain cellular homeostasis (Ma et al., 2020). Transcriptome analysis also identified 7 upregulated genes (*NKAIN4*, *ATP6V0D2*, *ATP6V1C2*, *ATP6V1G3*, *SLC4A8*, *KCNH1*, and *KCNMA1*) associated with the transport of Na^+^, K^+^, and H^+^ ions. Correlation analysis indicated these DEGs and eggshell phenotypic traits (eggshell thickness, eggshell strength, papilla structure, and uterine morphology) showed a significant positive correlation. These findings highlight the crucial role of dietary HF and ST in ion transport regulation and eggshell biomineralization.

Matrix proteins play a crucial role in the transition of calcium carbonate to stabilized calcite crystals, influencing the ultrastructural and mechanical properties of eggshells (Marie et al., 2015). The results of uterine RNA-Seq revealed that the HF+ST treatment significantly upregulated the transcription levels of genes encoding matrix proteins, such as *GPC4*, *MEPE*, *SPP1*, *CLU*, and *BPIFB3*. Glypicans (GPC) are implicated in biomineralization, with exclusive expression during eggshell calcification and close links to eggshell mechanical properties (Lavelin et al., 2002). Osteopontin (OPN), a glycosylated protein secreted by uterine epithelial cells, participates in forming the eggshell's organic matrix (Xin et al., 2021). *SPP1*, an osteoblast-encoding gene with a mineral-binding domain, is involved in calcium metabolism and calcium carbonate precipitation, with upregulated expression during eggshell mineralization ([Bibr bib45][Bibr bib49]). In vitro, calcite crystals containing OPN are harder than those without (Chien et al., 2008). OC-116, a matrix extracellular phosphate glycoprotein (MEPE), acts as a major eggshell matrix protein to regulate calcite crystal formation; together with ovocalyxin-32, it modulates eggshell elasticity and thickness (Sah et al., 2018). These upregulated matrix proteins may represent an important mechanism by which HF regulates eggshell microstructure and quality.

Kyoto Encyclopedia of Genes and Genomes pathway analysis revealed that the upregulated DEGs were mainly enriched in the MAPK signaling pathway, focal adhesion, and calcium signaling pathway. The MAPK pathway regulates key physiological processes such as cell growth, differentiation, apoptosis, and inflammation (Peter et al., 2003). The ERK 1/2 MAPK pathway has been shown to play an important role in the growth, development and differentiation of the uterus (Jeong et al., 2012). Moreover, elevated levels of anti-inflammatory factors reflect an effective host immune response in resolving inflammation (Zhang et al., 2022). In this study, the HF120+ST120 group exhibited significantly increased levels of the anti-inflammatory cytokines IL-4 and IL-10 in uterine tissues, suggesting an enhanced anti-inflammatory effect. Continued egg production and aging in older laying hens lead to oxidative stress, which impairs oviduct function (Wang et al., 2024). Excessive oxidative stress during eggshell formation causes uterine calcium translocation, ultimately reducing eggshell quality (Cao et al., 2024). MDA is a well-established biomarker of oxidative stress, while GSH-Px, T-AOC, and MDA are important indicators of antioxidant defense capacity ([Bibr bib45][Bibr bib47]). A meta-analysis of dietary plant extracts revealed that flavonoids, such as hesperidin and genistein, enhance serum GSH-Px and T-AOC activities while reducing MDA levels (Darmawan et al., 2022), which aligns with our findings. The results indicated that dietary supplementation with HF and ST may alleviate uterine inflammation and oxidative stress by activating the MAPK signaling pathway. Genes involved in the calcium signaling pathway have been shown to play a role in the transport of calcium and carbonate ions from the bloodstream to the uterus, regulating eggshell mineralization (Cheng et al., 2023). Further analysis revealed that the upregulation of ion transport-related genes (e.g., *CALB1* and *CLU*) was negatively correlated with uterine MDA levels and positively correlated with GSH-Px and IL-10 levels. Therefore, dietary HF and ST supplementation could alleviate the inflammation and oxidative stress induced by aging in the uterus, which improve eggshell mineralization.

Long-term laying behavior in older hens impacts oviduct microbial colonization, with these microbes playing a critical role in maintaining oviduct health (Cheng et al., 2024; Dai et al., 2023). Significantly higher α and β diversity were observed in the HF120+ST120 group compared to the CON group, indicating that the treatment altered species composition and enhanced the richness and evenness. Proteobacteria encompasses a broad range of pathogens, and its abundance is often considered a marker of ecological dysbiosis in animal microbiota ([Bibr bib41][Bibr bib42]). Increased Proteobacteria abundance in laying hens has been associated with elevated oxidative stress and reduced productivity (Da Rosa et al., 2019). Additionally, the increased abundance of Bacteroidetes and the decreased abundance of Proteobacteria improved the ability of laying hens to efficiently absorb and utilize nutrients (Guo et al., 2022), consistent with the results of the present study. *Ruminococcus* was shown to maintain intestinal pH by producing volatile fatty acids and butyrate, which inhibit the proliferation of pathogenic bacteria (Biasato et al., 2019; Zhang et al., 2022). Previous research has indicated that *Ruminococcus* species play a key role in promoting oviduct health in laying hens, particularly via their increased abundance in the uterus, which helps maintain epithelial barrier integrity and alleviate inflammatory damage (Li et al., 2024a). *Rikenellaceae_RC9_gut_group* is capable of fermenting indigestible carbohydrates to produce short-chain fatty acids (SCFAs), thereby improving the uterine microbial microenvironment and promoting calcium absorption ([Bibr bib41][Bibr bib43]). Flavonoids have been shown to enhance the health of laying hens by increasing the relative abundance of *Rikenellaceae_RC9_gut_group* (Liu et al., 2023a). In the present study, the relative abundances of uterine *Ruminococcus* and *Rikenellaceae_RC9_gut_group* were elevated in the HF120+ST120 treatment group. Therefore, the changes in uterine microbial composition induced by HF and ST treatments, along with subsequent improvements in microecology, may constitute a potential mechanism contributing to enhanced eggshell quality. The further analysis confirmed that Bacteroidota, *Ruminococcus*, and *Rikenellaceae_RC9_gut_groups* were positively correlated with the eggshell thickness, effective layer thickness, frequency of early fusion inmammillae, and eggshell fracture toughness. In contrast, the abundance of Proteobacteria was negatively correlated with eggshell effective layer thickness, eggshell fracture toughness, and frequency of early fusion of papillae, but positively correlated with the ratio of mammillary layer and the number of B-type mammillae. The abundance of *Ruminococcus* and *Rikenellaceae_RC9_gut_group* was negatively correlated with the ratio of mammillary layer and the number of B-type mammillae. In addition, the abundance of Bacteroidota, *Ruminococcus*, and *Rikenellaceae_RC9_gut_group* was positively correlated with IL-10 levels in uterine tissues and negatively correlated with MDA levels. The abundance of *Rikenellaceae_RC9_gut_group* and Bacteroidota was positively correlated with T-AOC levels in uterine tissues, whereas the abundance of *Mycoplasma* and Proteobacteria was negatively correlated with these levels. Finally, the abundance of Bacteroidetes was positively correlated with IL-4 levels in uterine tissues, while that of Proteobacteria was negatively correlated with IL-10 levels. Thus, dietary supplementation with HF and ST may help balance oviductal homeostasis in laying hens, thereby improving eggshell quality.

## 5 Conclusions

Dietary supplementation with HF and ST improved the microstructural and mechanical properties of eggshells in laying hens, with the optimal results observed at a combined doses of HF 120 mg/kg and ST 120 mg/kg. Enhanced eggshell quality was strongly correlated with uterine tissue morphology, calcium transport, matrix protein synthesis, antioxidant and anti-inflammatory responses, as well as microbial homeostasis. Therefore, the combination of HF and ST demonstrates synergistic effects, effectively promoting oviduct health and eggshell quality during the late laying period.

## Credit Author Statement

**Jiao Wang:** Writing – original draft, Visualization, Methodology, Investigation, Formal analysis, Data curation, Conceptualization. **Qiang Jiang:** Writing – review & editing, Visualization, Investigation, Formal analysis. **Xianlei Li:** Writing – review & editing, Validation, Methodology. **Xianghong Zhao:** Writing – review & editing, Visualization, Methodology. **Yuming Guo:** Resource. **Bingkun Zhang:** Resources, Investigation. **Zhonghua Ning:** Resource. **Zengpeng Lv:** Writing – review & editing, Supervision, Resources, Funding acquisition.

## Declaration of competing interest

We declare that we have no financial and personal relationships with other people or organizations that can inappropriately influence our work, and there is no professional or other personal interest of any nature or kind in any product, service and/or company that could be construed as influencing the content of this paper. The authors declare the following financial interests/personal relationships which may be considered as potential competing interests: Xianghong Zhao is currently employed by Beijing SinoAgri Uplus Biotech Co., Ltd.
