# Supplementary material for: Regulatory effects of hawthorn leaf flavonoids and stevioside on the uterine function and eggshell quality in laying hens
Source: Anim Nutr. 2025 Aug 26;23:191–205. doi: 10.1016/j.aninu.2025.06.007 (PMC12664048; doi:10.1016/j.aninu.2025.06.007)
Supplement: Multimedia component 1 [file mmc1.docx]

**Table S1** Primer sequences used for quantitative real-time PCR assay.

| Genes | Full names | Primer sequences (5’–3’) | Accession no. |
| --- | --- | --- | --- |
| *β-Actin* | Beta-Actin | F:GAGAAATTGTGCGTGACATCA  R:CCTGAACCTCTCATTGCCA | L08165 |
| *CALB1* | Calbindin 1 | F:TGTTATGGAGTGCAGGATGG  R:TAGAGCGAACAAGCAGGTGA | NM-205513.1 |
| *ATP2B1* | ATPase plasma membrane Ca^2+^ transporting 1 | F:CTGCACTGAAGAAAGCAGATGTTG  R:GCTGTCATATACGTTTCGTCCCC | XM-416133 |
| *ATP2B2* | ATPase plasma membrane Ca^2+^ transporting 2 | F:TTACTGTACTTGTGGTTGCTGTCCC  R:GGTTGTTAGCGTCCCTGTTTTG | XM-001231767 |
| *SLC8A1* | Solute carrier family 8member A1 | F:TCACCTTCTTCTTCTTCCCAATCT  R:GCAACCTTTCCGTCCATCTC | NM 001079473.1 |
| *SLC8A3* | Solute carrier family 8 member A3 | F:GGAGAGACCACAACAACAACCATTC  R:AGCTACGAATCCATGCCCACAC | XM_425326.6 |
| *ATP2A3* | ATPase endoplasmic reticulum Ca^2+^ transporting 3 | F:CAACCCCAAGGAGCCTCTTATC  R:GGTCCCTCAGCGTCATACAAGAAC | XM_021270084.2 |
| *SLC4A8* | Solute carrier family 4 member 8 | F:GATGGTGGAGCCGATGTGAG  R:CCAGCTCCTCCTTCTCATAGT | NM_205262.1 |
| *ATP6V1G3* | ATPase H^+^ transporting V1 subunit G3 | F:CTCAGCTAGAGGAGCAAACAA  R: GACTTCGGGCTTGACATCATA | XM_422192.4 |
| *ATP6V1C2* | TPase H^+^ transporting V1 subunit C2 | F: ACCAAACAGGAAGTCGGTAAA  R: GTCCATGCCAGGATCCATTAT | XM_025148940.1 |
| *SPP1* | Secreted phosphoprotein 1 | F:AGGTGGACGGAGGAGACA  R:ACGGGTGACCTCGTTGTT | NM-204535.5 |
| *BPIFB3* | BPI fold containing family B member 3 | F:CCTGAAGCCACACCTCACTAAGC  R:ATCGCCAACAGTCCCAACAAGATC | XM_046930818.1 |

F = **forward**; R = reverse.

**Table S2** RNA-seq statistics for 12 samples.

| Samples^1^ | Raw reads, bp | Clean reads, bp | Mapped reads, % | GC Content, % | Q30, % |
| --- | --- | --- | --- | --- | --- |
| A1 | 46,619,814 | 45,956,992 | 92.47 | 50.29 | 96.07 |
| A2 | 37,469,322 | 36,929,210 | 93.30 | 49.62 | 96.08 |
| A3 | 53,042,162 | 52,171,174 | 92.20 | 50.94 | 95.90 |
| A4 | 49,328,516 | 48,599,854 | 93.35 | 49.41 | 96.03 |
| A5 | 60,343,106 | 59,352,032 | 92.40 | 50.52 | 95.79 |
| A6 | 64,751,572 | 63,690,712 | 92.71 | 50.95 | 95.83 |
| D1 | 47,538,390 | 46,625,198 | 92.33 | 49.80 | 95.40 |
| D2 | 62,816,780 | 61,662,074 | 91.04 | 52.01 | 95.08 |
| D3 | 49,195,230 | 48,296,646 | 92.68 | 49.46 | 95.60 |
| D4 | 50,663,792 | 49,715,268 | 93.44 | 47.49 | 95.35 |
| D5 | 55,441,252 | 54,236,896 | 92.21 | 50.48 | 95.19 |
| D6 | 58,870,924 | 57,793,828 | 90.70 | 50.49 | 95.75 |

^1^A1-A6 represent the HF120+ST120 group, D1-D6 represent the CON group. CON, basal diet; HF120+ST120, basal diet supplemented with 120 mg/kg HF and 120mg/kg ST.
